# Clusters of Patient Empowerment and Mental Health Literacy Differentiate Professional Help‐Seeking Attitudes in Online Mental Health Communities Users

**DOI:** 10.1111/hex.70153

**Published:** 2025-01-15

**Authors:** Nicole Bizzotto, Gert‐Jan de Bruijn, Peter Johannes Schulz

**Affiliations:** ^1^ Faculty of Communication, Culture and Society Università della Svizzera italiana Lugano Switzerland; ^2^ Department of Communication Studies University of Antwerp Antwerpen Belgium; ^3^ Department of Communication & Media Ewha Womans University Seoul South Korea

**Keywords:** help‐seeking attitudes, mental health literacy, online health communities, patient empowerment

## Abstract

**Objectives:**

Grounded in the Health Empowerment Model, which posits that health literacy and patient empowerment are intertwined yet distinct constructs, this study investigates how the interplay of these factors influences attitudes toward seeking professional psychological help in members of online communities for mental health (OCMHs). This while acknowledging the multidimensionality of patient empowerment, encompassing meaningfulness, competence, self‐determination, and impact.

**Design and Methods:**

A cluster analysis of data gathered from 269 members of Italian‐speaking OCMHs on Facebook has been performed.

**Results:**

Four profiles have been identified: dangerous self‐managers (11.2%), effective self‐managers (21.2%), disempowered (40.5%) and ambivalent empowered (27.1%). Clusters provided meaningful variations in help‐seeking attitudes, also when controlling for depression and anxiety severity, *F*_3, 265_ = 11.910, *p* < 0.001.

**Conclusions:**

The findings provided further evidence of the multidimensionality of patient empowerment. Considering the results, we discussed potential interventions aimed at enhancing the quality of OCMHs, tailoring to the unique characteristics of each cluster.

**Patient or Public Contribution:**

Administrators and moderators of mental health Facebook communities—whether expert‐led by mental health professionals or peers—played a key role in this study. They provided valuable insights during the questionnaire design process to ensure the questions were both relevant and appropriate for community members. These administrators and moderators also actively facilitated participant recruitment by creating and sharing posts, either video‐ or text‐based, on community homepages. Furthermore, after completing the questionnaire, participants were encouraged to comment on the Facebook posts where the survey link was shared, mentioning that they participated and inviting other members to take part. This approach aimed to foster a sense of involvement and further promoted the survey within the community.

## Summary

**What is already known on this subject?:**

- Health literacy and (multidimensional) patient empowerment are intertwined yet distinct constructs.
- Individuals suffering from mental health disorders are increasingly using online communities.
- Lower mental health literacy is associated with less favourable attitudes towards seeking help.

**What does this study add?:**

- A novel approach (cluster analysis) to test the Health Empowerment Model.
- The interaction of literacy and multidimensional empowerment provides variations in help‐seeking attitudes.
- Discussion on clusters' targeted interventions to ameliorate online mental health communities utilization.

## 1 Introduction

The landscape of mental healthcare has undergone a profound transformation in recent decades. Until the late 20th century, the dominant mental healthcare paradigm heavily relied on a paternalistic approach [1] where people with mental health conditions were disempowered due to poor information provision and coercive treatment conditions [2, 3]. Although the power imbalance of patients vis‐à‐vis mental health providers is still considered greater than in other healthcare sectors [4], in contemporary times there has been a significant shift to more collaborative models of healthcare delivery [5]. As a result, patients are increasingly asked to voice their opinion [6, 7]. From their perspective, patients also express a desire to participate in healthcare decisions [8]. Therefore, patients' knowledge of health matters has recently become more important than ever.

This shift is also thought to be a consequence of what has recently been defined as the ‘digital revolution’ in mental healthcare [9]. In the words of Calvillo et al. [142], ‘technology is empowering patients' as the digital landscape has provided individuals with unprecedented access to information, facilitating greater patient empowerment and contributing to a move away from paternalism.

While research in this domain predominantly centred on identifying the factors that predict online health information‐seeking behaviour [10, 11], there is a noticeable gap when it comes to understanding the diverse profiles of individuals performing the behaviour, for instance, within online health communities. Generally, studies tend to rely on the theoretical framework of the Uses and Gratifications to understand media usage and access according to which individuals actively seek out media to gratify specific wants and needs [12, 13] such as information and social connection [14].

However, it is crucial to consider that individuals should also be distinguished based on their ability to critically evaluate the online content and whether they intend to utilize the information as a substitute or as a supplement to professional help. This is particularly important given that individuals experiencing greater health challenges are more likely to actively seek and rely on online health information [15].

One way in which individuals suffering from a mental health disorder can find information to understand their condition [16], receive group‐level support [17] and evaluate possible treatments is through the use of online communities, that are ‘available 24 hours per day, 7 days per week and draw together a heterogeneous mixture of people' [18]. Within these communities, becoming increasingly popular [19, 20], members often simultaneously seek advice and provide support [21].

These groups are increasingly present on social media platforms [20] where not all information is deemed to be reliable [22], and members may struggle to retrieve it or use it correctly. As a result, we are now witnessing the emergence of highly empowered patients with varying levels of health literacy, and potentially exposed to a vast amount of inaccurate information online.

If, on one side, empowerment is traditionally regarded as a favourable outcome of participation in online communities [23], when combined with lower levels of literacy, it can sometimes align with tendencies to self‐reliance, considered as a primary barrier to professional help‐seeking [24, 25, 26]. Furthermore, a recent concept analysis of health information–seeking behaviour suggests that the desire for empowerment may motivate individuals to participate in those communities [27], where they often seek information not only to better understand their health but also to self‐diagnose [28].

This paper delves into the complex interplay between health literacy and empowerment in online mental health communities, exploring whether this combination of constructs influences attitudes towards professional help‐seeking—a pivotal concept in predicting health behaviour [29].

## 2 Literature Review

### 2.1 The Health Empowerment Model

The Health Empowerment Model [34] has proven to be a cogent framework in explaining differences in healthcare‐related behaviours [30, 31, 32]. The model considers health literacy and patient empowerment as independent yet equally important determinants of health outcomes such that a sense of autonomy in managing one's health status (patient empowerment) does not necessarily equate with actual competencies, as they depend on other aspects such as health literacy.

### 2.2 Patient Empowerment

The first aspect of the model, that is, patient empowerment refers to a multidimensional construct that has its origins in the workplace setting, where its pioneering researcher, Spreitzer, developed its four key components: Meaningfulness, Competence, Self‐determination, And Impact [33]. *Meaningfulness* pertains to an individual's personal relevance regarding a specific task, *Competence* focuses on their perceived ability to perform the task, *Self‐determination* encompasses the sense of causal responsibility for one's actions, emphasizing autonomy and the ability to make independent choices. Finally, the dimension of *Impact* underscores the perceived contribution to accomplishing the task.

Subsequently, these components were adopted in the health communication context by Schulz and Nakamoto [34, 143] as components of patient empowerment, a critical determinant of improved health status [5]; that is, self‐determination refers to the degree to which individuals think that their health choices are determined by themselves. Empowerment in the health context is defined as patient empowerment ‘despite the fact that persons who are not at the moment patients are also included when for instance disease prevention or the position of individuals vis‐à‐vis the health system are treated’ [34, p. 5]. Consequently, we will use the term patient empowerment regardless of whether study participants were mental health clients at the time of data collection.

### 2.3 Health Literacy and Mental Health

The Health Empowerment Model [34] classifies individuals with high levels of empowerment into two distinct groups: *Dangerous self‐managers* and *Effective self‐managers*, based on their respective levels of health literacy, where the former exhibits low levels, while the latter has high literacy levels. Similarly, the model categorizes individuals with low patient empowerment into *Needlessly dependent patients* (high literacy) and *High‐needs patients* (low literacy).

Concerning health literacy, there are numerous definitions [35] that share common core elements such as the skills that enable individuals to obtain, understand, appraise and use information to make decisions and take actions that will have an impact on health status [36]. Health literacy has been recognized as a critical determinant of health in the World Health Organization Shanghai Declaration [144] and is considered an increasingly important construct, especially in the online context where people can frequently stumble upon misinformation [145, 146, 147].

Literacy of the declarative type (factual knowledge related to health issues; see [37]) is especially important in the mental health field, where knowledge about symptoms is necessary to seek appropriate care when needed [38, 39, 40]. The concept of mental health literacy has arisen from the domain of health literacy, and it is defined as knowledge about mental health disorders that is associated with their recognition, management and prevention [41]. In a recent review, Furnham and Swami [41] found the general public has relatively poor recognition of mental health with a tendency to rely on self‐help over traditional professional treatments.

## 3 The Present Study

### 3.1 Research Context: Italian Online Communities for Mental Health on Facebook

Mental health problems represent one of the leading causes of societal burden [148]. Given the stigma surrounding mental health–related issues, patients are likely to turn to online resources such as online mental health communities (OCMHs) for support [20, 42, 43].

In the Italian context, there is a prevailing issue with patients receiving inadequate information about mental health concerns [44]. Additionally, when compared to other high‐income nations, Italy has fewer professional resources for mental health [45] and lower levels of health literacy [46].

The present study concentrates on the Facebook platform as although the total number of Facebook users has plateaued in recent years [47], the number of users who are meaningfully engaged in Facebook groups has quadrupled [48]. Online communities are, in fact, one of the most popular features of the social networking site [49]. Lastly, there is a vast amount of Facebook mental health communities in the Italian language, offering a unique setting for our investigation. Our focus on a single country aligns with the approach taken in other studies, such as those conducted in Lithuania [50] and France [51].

### 3.2 Help‐Seeking Attitudes

With respect to help‐seeking, scholars distinguish between formal assistance, provided by mental health professionals or informal support [52]. Henceforth, when we refer to ‘help‐seeking’, we will specifically refer to formal assistance.

In the mental health context, help‐seeking attitude is defined as individuals' perceptions or beliefs regarding seeking psychological help [53]. Several studies have revealed the predictive importance of attitudes in the help‐seeking process [54, 55, 56]. Fischer and Turner [149] first theorized that one's attitude towards help‐seeking underpins actual help‐seeking mental health behaviour. To assess this, the two authors (1970) developed the widely used Attitudes Toward Seeking Professional Psychological Help scale, which we have also used in the present study.

Help‐seeking attitudes are a particularly important aspect to investigate as a substantial amount of people avoid help‐seeking for mental health concerns [57]. Also, people tend to think that mental health difficulties will resolve themselves spontaneously [58] and there is a preference to manage the problem on their own [59, 60, 61]. This is concerning as early help‐seeking for mental health issues has been shown to promote early intervention and improved long‐term outcomes [140]. Previous systematic reviews have shown that help‐seeking in the mental health context can be attributed to multiple factors, including emotional competence, stigma, mental health literacy, social support, self‐reliance and lack of accessibility (e.g., time, transport, cost) [24, 62]. In addition to key themes related to the macro‐categories of knowledge (stigma, mental health literacy, knowledge about health services) and patient empowerment (e.g. self‐reliance)—research has consistently shown that past experiences with mental health services play a critical role in shaping attitudes and intentions towards seeking professional support. This pattern was observed across various age groups, including children and adolescents [24, 63] and older adults [64], and diverse populations such as Chinese [25], Filipino [65], Ethiopian [66], and within males [67].

### 3.3 Study Aims

To the best of our knowledge, no previous research has performed cluster analysis on the interplay between patient empowerment and health literacy. Instead, previous studies have predominantly relied on median splits or explored health literacy as a moderator of empowerment, often focusing on other health conditions such as chronic low back pain and fibromyalgia [68, 69, 70]. Cluster analysis, however, can offer a more granular depiction and a nuanced approach to understanding the interaction of the two constructs.

Previous studies on online health communities have treated empowerment as an outcome of online health community participation [23, 71, 72, 73, 74, 75]. However, levels of empowerment can significantly vary among individuals in online contexts [76, 77, 78], a pattern similarly observed in the context of health literacy [79, 80]. Consequently, our study aims to differentiate patients based on their levels of literacy and patient empowerment, focusing on the potential differences in help‐seeking attitudes that may arise from the interaction of the two constructs:

Hypothesis 1 Profiles created from the interaction of mental health literacy and patient empowerment will differ in help‐seeking attitudes.

The cluster analysis approach is inherently exploratory though, guided by the Health Empowerment Model [34], we anticipate finding several different profiles of OCMHs, that might include one characterized by high empowerment and low literacy, and another with high empowerment and high literacy. We also expect to identify at least one group with lower empowerment levels, in line with previous studies in online communities [76, 77, 78].

In the present study, we decided to differentiate depression literacy, misinformation agreement and agreement with correct information as we believe that one thing is knowledge of depression, another is being illiterate to general mental health knowledge, including agreeing with beliefs that might prevent help‐seeking.

Concerning the potential impact of these clusters on help‐seeking attitudes, multiple prior studies have demonstrated that lower mental health literacy is associated with less favourable attitudes towards seeking help [81, 82, 83, 84, 85, 86] but never taking into account the role that patient empowerment might play. To assess the differences in attitudes across clusters, after inspecting differences in mental health symptomatology across the clusters, we will also control for depression and anxiety severity, given their prevalence in Italy [87], and as previous research has shown a significant correlation between these mental illnesses and help‐seeking attitudes [81, 88].

In line with previous studies ([61, 62, 150, 151, 152, 153]; for a review, see Gulliver et al. [24]), we also expect clusters with higher levels of self‐determination empowerment, a concept related to self‐reliance, to have more negative help‐seeking attitudes. Thus, given the multidimensionality of patient empowerment [34], we are also interested in exploring whether:

Hypothesis 2 Within each specific group identified through cluster analysis, there will be variability in the levels of the various dimensions of patient empowerment, that is, Meaningfulness, Competence, Self‐determination, and Impact.

In other words, having high levels in one dimension does not inherently equate to higher levels in the other dimensions.

## 4 Methods

### 4.1 Measures

#### 4.1.1 Mental Health Literacy

We assessed literacy with three different variables. First, we used the Italian‐validated version of the Depression Literacy Questionnaire, which assesses mental health literacy specific to depression [89]. The questionnaire consists of 22 items. Respondents can answer each item with one of three options (true, false, or don't know) and each correct response receives one point. Higher scores indicate higher mental health literacy of depression. In addition, recognizing the prevalence of misinformation in online health communities, we included two measures, namely ‘Agreement with misinformation’ and ‘Agreement with correct information’. Items were built from a previous content analysis in OCMHs [90, 91]. Both measures comprised three items, assessed using a Likert scale ranging from 1 (completely disagree) to 7 (completely agree), with the index score calculated as the average of all item ratings.

#### 4.1.2 Patient Empowerment

The scale of patient empowerment in the context of OCMHs was built based on Spreitzer [33] and previous studies on the Health Empowerment Model [68, 70]. Participants responded to a list of 10 items on a Likert scale ranging from 1 (strongly disagree) to 7 (strongly agree) with higher values suggesting higher levels of empowerment. Principal component analysis with the varimax rotation method was performed to identify the different dimensions of empowerment. See the Appendix for the items' list.

#### 4.1.3 Attitudes Towards Seeking Psychological Help‐Short Form (ATSPPHS‐SF)

The ATSPPHS‐SF consists of 10 items on a scale from 1 (*strongly disagree*) to 6 (*strongly agree*). Higher scores represent more positive attitudes. We used the Italian‐validated version [92].

#### 4.1.4 Center for Epidemiological Studies‐Depression (CES‐D)

The CES‐D Scale [93] is a 20‐item self‐report inventory developed to measure depressive symptoms. Participants report the frequency of experiencing symptoms within the past week on a scale ranging from 0 (*rarely*) to 3 (*most or all of the time*). Scores range from 0 to 60, with high scores indicating greater depressive symptoms. The scale has been validated in the Italian language [94].

#### 4.1.5 General Anxiety Disorders (GAD‐7)

The seven‐item GAD has been developed to identify individuals with a generalized anxiety disorder [95]. Participants indicated agreement with the presence of symptoms such as ‘Feeling nervous, anxious or on edge’ on a Likert scale ranging from 0 (*not at all*) to 3 (*nearly every day*). The total score ranges from 0 to 21. We made use of the Italian‐validated version [96].

### 4.2 Procedure

Data for this study were collected through a Qualtrics survey during spring 2022. To recruit participants, the principal investigator reached out to administrators of 72 Italian‐speaking OCMHs, identified by entering mental health–related keywords into the Facebook search bar. In total, 65% of OCMHs (*N* = 51) agreed to participate, while 12% (*N* = 9) declined, and 15% (*N* = 12) did not respond. Administrators and moderators of the OCMHs were invited to complete the survey (responses excluded from the analysis) both to familiarize themselves with the content being shared within their communities and to actively engage in the process, in line with the Patient Involvement and Engagement principles [97].

Participants were recruited through a video presentation shared on the collaborating OCMHs, including a link to the questionnaire. Administrators and moderators also actively facilitated participant recruitment by creating and sharing posts, either video‐ or text‐based, on community homepages. Upon providing informed consent, participants were asked to complete sections on socio‐demographic variables, mental health literacy and help‐seeking attitudes. After completing the questionnaire, participants were encouraged to comment on the Facebook posts where the survey link was shared, mentioning that they participated and inviting other members to take part. This approach aimed to foster a sense of involvement and further promoted the survey within the community. In the span of 2 or 3 days, participants were contacted through their preferred communication method, be it email, WhatsApp, or Facebook Messenger, and provided with a link to the second segment of the survey, which included questions related to empowerment and depression and anxiety severity. On average, participants completed the second questionnaire 4 days later. This procedure was implemented to facilitate the data collection process. Participants were rewarded with a financial incentive (lottery for seven vouchers worth 50 euros each) and access to artistic/poetry videos. In total, we collected 276 responses with all variables of the present study filled out. Before applying listwise deletion, we had collected 604 participants for the first part of the survey and 285 participants for the second part. Furthermore, seven responses were eliminated from the data set as participants were using the OCMHs only as informal caregivers. The Ethics Committee of Università della Svizzera Italiana approved the study design.

## 5 Data Analysis

### 5.1 Preliminary Analyses

The normality of the variables was evaluated by inspecting the skewness and kurtosis. The Mahalanobis distance [154] was used to determine multivariate outliers (statistical significance 0.001). We calculated McDonald's omega reliability coefficient (ω). When variables had less than four items, we reported also the inter‐item correlation range (three items) or Pearson correlation (two items) as suggested by other authors [98].

### 5.2 Cluster Analysis

Cluster analysis classifies into meaningful, mutually exclusive groups based on similarities with respect to selected variables [99], ‘so that members of the resulting groups are as similar as possible to others within their group (high within‐group homogeneity) and as different as possible to those in other groups (low between‐group homogeneity)’ [100, p. 330].

In Step 1 we performed a hierarchical cluster analysis with the R package *NbClust* [101]. As an agglomeration method, we used ‘centroid’, considered as more robust than others in terms of isolated points [102]. The distance metric was set to ‘Maximum’ (Chebyshev distance [103]). The optimal number of clusters (*k*) is determined by the majority principle (i.e., the solution with the most indices in favour) and theoretically meaningful solutions.

### 5.3 Inferential Tests

In Step 2, before conducting inferential tests, Levene's homogeneity of variance tests were performed. Following this, we employed one‐way analysis of variance tests (ANOVAs) to examine differences in the clusters for the variables defining clusters, mental health symptomatology and help‐seeking attitudes. In case of significance, ANOVAs were followed by Tukey's honestly significant difference (HSD) test for multiple comparisons or in case of violation of homogeneity of variance, by post‐hoc Games‐Howell. Eta squared (η²) was used to determine effect size. We then performed a non‐parametric analysis of covariance (ANCOVA) using Quade's test to compare the coefficients of help‐seeking attitudes (dependent variable) among the various clusters (independent variable) adjusting for disease severity (covariates).

## 6 Results

**Figure 1:**
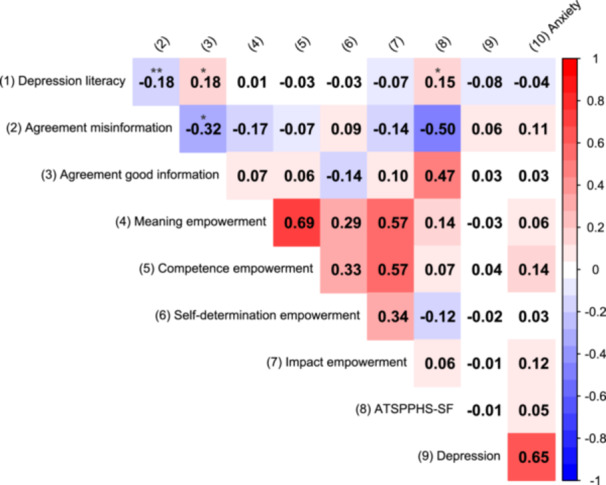
Heatmap of Pearson's correlations. *p < 0.05, **p < 0.01, ***p < 0.001.

The values of skewness and kurtosis of all the variables in this study are within the acceptable range (see Table 2). No multivariate outliers were found. In Figure 1, we present the heatmap of the correlations among variables.

### 6.1 Sample Characteristics

Participants ranged from 20 to 76 years old (*M*_age_ = 42.65, SD_age_ = 11.23). Socio‐demographic details are presented in Table 1. Considering the strictest CES‐D cut‐off of 20 [155], the majority of participants likely suffered from depression (190/269; 71%). A less pronounced trend holds true also for anxiety using a cut‐off of 10 [104] with 58% (156/269) of members likely suffering from generalized anxiety disorder.

**Table 1:** Socio‐demographics (*N* = 269).

	*N*	%
Gender		
Male	40	14.9
Female	227	84.4
Other	1	0.4
Prefer not to respond	1	0.4
Education		
Middle school or under	36	12.6
High school	143	53.2
University degree	83	30.8
Doctorate	7	2.6
Employment		
Employed	146	54.3
Unemployed	71	26.4
Retired	13	4.8
Disabled, unable to work	18	6.7
Student	21	7.8
Perceived economic status		
Very bad	75	27.9
Fair	148	55.0
Good	41	15.2
Very good	5	1.9
Current treatment		
Psychological therapy	37	13.8
Pharmacological therapy	66	24.5
Both psychotherapy and pharmacological therapy	99	36.8
Currently not in therapy	59	21.9
Prefer not to answer	8	3.0

**Table 2:** Descriptive statistics (*N* = 269).

	Mean	SD	Skewness	Kurtosis	ω/ICC/*r*
Depression literacy (1–22)	14.45	3.35	−0.33	−0.15	0.70
Agreement with misinformation (1–7)	2.31	1.19	1.01	0.62	0.60/0.17–0.40
Agreement with correct information (1–7)	6.44	.92	−2.02	4.05	0.59/0.07–0.54
Meaning empowerment (1–7)	4.10	1.57	−0.21	−0.52	0.911/0.75–0.79
Competence empowerment (1–7)	3.73	1.46	0.13	−0.52	0.821/0.54–0.56
Self‐determination empowerment (1–7)	2.29	1.51	1.21	−0.67	0.528
**I**mpact empowerment (1–7)	4.39	1.54	−0.28	−0.47	0.418
ATSPPHS‐SF (1–6)	4.82	0.75	−1.12	2.54	0.770
Depression severity (0–60)	30.21	15.27	−0.01	−1.05	0.930
Anxiety severity (0–21)	11.58	5.65	0.02	−1.17	0.897

### 6.2 Clusters

According to the majority rule and theoretically sound solution, *k* = 4 (*N* = 7) was appropriate, although *k* = 2 was the most proposed (*N* = 9), followed by *k* = 3 (*N* = 4), and *k* = 8 (*N* = 3).

Clusters 1 and 2 have been named according to the Health Empowerment Model [105]. In response to reviewer concerns, we wish to clarify that terms such as ‘dangerous’ and ‘effective’ describe potential behaviours that individuals may exhibit, rather than characteristics of individuals. This approach is consistent with trauma‐informed principles of empowerment, choice and control [106], as the term ‘dangerous’ is used to describe a behavioural pattern that emerges from the combination of high empowerment and low literacy. We refrain from judging why this pattern could have arisen, nor are we seeking to demonize it, as individual and collective empowerment are crucial for mental health recovery [107, 108]. See Table 3 for descriptive statistics of clusters' means.

**Table 3:** Descriptives of cluster means.

	Cluster 1: Dangerous Self‐managers	Cluster 2: Effective Self‐managers	Cluster 3: Disempowered	Cluster 4: Ambivalent empowered
	*N* = 30 (11.2%)	*N* = 57 (21.2%)	*N* = 109 (40.5%)	*N* = 73 (27.1%)
	*M* (SD)	*M* (SD)	*M* (SD)	*M* (SD)
Variables defining clusters				
Depression literacy	13.17 (2.52)	17.10 (2.27)	15.47 (2.74)	11.40 (2.59)
Agreement with misinformation	4.23 (1.20)	1.73 (0.74)	2.02 (0.93)	2.41 (1.02)
Agreement with correct information	4.94 (1.34)	6.71 (0.65)	6.63 (0.62)	6.56 (0.71)
Empowerment Meaning	3.16 (1.46)	5.29 (1.08)	3.08 (1.26)	5.10 (0.99)
Empowerment Competence	3.14 (1.02)	4.85 (1.14)	2.67 (1.03)	4.70 (1.08)
Empowerment Self‐determination	2.98 (1.65)	3.66 (1.85)	1.41 (0.65)	2.25 (1.11)
Empowerment Impact	3.50 (1.12)	5.54 (0.94)	3.39 (1.28)	5.35 (1.17)
Auxiliary variables				
Attitudes (1–6)	4.13 (1.31)	5.18 (0.76)	5.11 (0.67)	5.14 (0.64)
Anxiety	12.03 (5.44)	11.09 (5.56)	10.84 (5.58)	12.86 (5.80)
Depression	32.37 (13.97)	28.16 (14.49)	29.77 (15.83)	31.60 (15.60)

#### 6.2.1 Cluster 1: Dangerous Self‐Managers

Cluster 1 represents about 11.2% of members. It is characterized by the second‐lowest rate of depression literacy and with significantly higher agreement with misinformation when compared to all other categories (*p* < 0.001). With respect to Meaning, Competence and Impact empowerment, levels fall below the average of all members, significantly differing from those of Clusters 2 and 4 (*p* < 0.001). With respect to Self‐determination, there was a significant difference with Cluster 3 (mean difference [MD] = 1.57, *p* < 0.001).

#### 6.2.2 Cluster 2: Effective Self‐Managers

Cluster 2 represents about 21.2% of members and exhibits the highest rate of depression literacy compared to the other clusters. Members significantly agreed less with misinformation compared to Clusters 1 (MD = −2.50, *p* < 0.001) and 4 (MD = −0.68, *p* < 0.001) and their levels of empowerment were significantly higher than those of all the other clusters (*p* < 0.001) except for a nonsignificant difference in Meaning, Competence and Impact with Cluster 4 and in Self‐determination with Cluster 1.

#### 6.2.3 Cluster 3: Disempowered

Approximately two‐fifths of the members belong to Cluster 3. They have the second‐highest depression literacy, behind the Effective self‐managers (*p* < 0.001). With respect to agreement with misinformation, they significantly differed from Dangerous self‐managers (MD = −2.21, *p* < 0*.*001) and Ambivalent empowered (MD = −0.34, *p* = 0.04) Overall, they have the lowest levels of empowerment compared with all the other clusters except from Cluster 1 from which they do not significantly differ in term of Meaning, Competence and Impact.

#### 6.2.4 Cluster 4: Ambivalent Empowered

**Figure 2:**
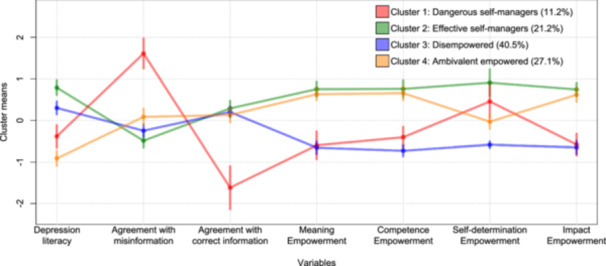
Cluster means with 95% confidence intervals. Clustering was performed on standardized data.

Cluster 4 (27.1% of participants) displays the lowest level of depression literacy, which is even lower than that observed in the Dangerous self‐managers (MD = −1.77, *p* = 0.01). Their level of agreement with misinformation, while significantly lower than that of the Dangerous self‐managers (MD = −0.21, *p* < 0.001), is significantly higher than the Disempowered (MD = 0.34, *p* = 0.04) and the Effective self‐managers (MD = 0.68, *p* < 0.001). However, compared to these last two clusters, no differences were observed in agreement with correct information. In terms of Meaning, Competence and Impact empowerment, they do not differ significantly from Effective self‐managers, from which instead, they differ (MD = −1.42, *p* < 0.001) in terms of Self‐determination (see Figure 2).

### 6.3 Differences in Help‐Seeking Attitudes and Mental Health Symptomatology Across the Clusters

**Figure 3:**
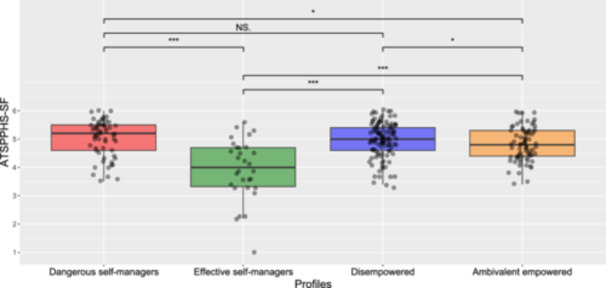
Output of the ANCOVA (Quade's test). *p < 0.05, ***p < 0.001, NS = nonsignificant.

With respect to differences in help‐seeking attitudes between the groups, the significant ANOVA (*F*_3, 265_ = 19.673, *p* < 0.001, η^2^ = 0.182) was followed by a Games‐Howell test of multiple comparisons that found that Dangerous self‐managers had significantly (*p* < 0.001) more negative help‐seeking attitudes compared to all the other clusters (see Table 4). With respect to depression and anxiety severity, the ANOVA resulted in no statistically significant differences between groups for depressive (*F*_3, 265_= 0.773, *p* = 0.510) and anxiety symptomatology (*F*_3, 265_ = 2.101, *p* = 0.100). After controlling for depression and anxiety severity, the pairwise comparisons (Games‐Howell test) of the significant ANCOVA using Quade's test (*F*_3, 265_ = 11.910, *p* < 0.001) were slightly different from the previous ANOVA (see Figure 3). As for the ANOVA, Cluster 1 was significantly different from all the other clusters (*p* < 0.001). In addition, Cluster 4 had significantly less favourable help‐seeking attitudes compared with Cluster 2 (*p* = 0.027) and 3 (*p* = 0.032).

**Table 4:** Inferential tests.

Variable	Levene's test, *p*‐value	One‐way ANOVA test, *p*‐value (*df* = 3, 265), η^2^	Tukey HSD post hoc pattern	Games‐Howell post hoc pattern
Variables defining clusters				
Depression literacy	0.796, *p* = 0.497	62.174, *p* < 0.001, 0.413	1 < 2***; 1 < 3***; 1 > 4**; 2 > 3***; 2 > 4***; 3 > 4***	/
Agreement with misinformation	2.80, *p* = 0.040	51.390, *p* < 0.001, 0.368	/	1 > 2***; 1 > 3***; 1 > 4***; 2 = 3 ns; 2 < 4***; 3 < 4*
Agreement with correct information	1.393, *p* < 0.001	43.966, *p* < 0.001, 0.332	/	1 < 2***; 1 < 3***; 1 < 4***; 2 = 3 ns; 2 = 4 ns; 3 = 4 ns
Empowerment Meaning	3.07, *p* = 0.029	7.668, *p* < 0.001, 0.444	/	1 < 2***; 1 = 3 ns; 1 < 4***; 2 > 3***; 2 = 4 ns; 3 < 4***
Empowerment Competence	0.396, *p* = 0.756	79.812, *p* < 0.001, 0.475	1 < 2***; 1 = 3 ns; 1 < 4***; 2 > 3***; 2 = 4 ns; 3 < 4***	/
Empowerment Self‐determination	34.81, *p* < 0.001	45.048, *p* < 0.001, 0.338	/	1 = 2 ns; 1 > 3***; 1 = 4 ns; 2 > 3***; 2 > 4***; 3 < 4***
Empowerment Impact	2.728, *p* = 0.045	66.880, *p* < 0.001, 0.431	/	1 < 2***; 1 = 3 ns; 1 < 4***; 2 > 3***; 2 = 4 ns; 3 < 4***
Auxiliary variables				
Attitudes	6.0671, *p* < 0.001	19.673, *p* < 0.001, 0.182	/	1 > 2***; 1 = 3 ns; 1 = 4 ns; 2 < 3***; 2 < 4***; 3 = 4 ns
Anxiety	0.566, *p* = 0.638	2.101, *p* = 0.100	/	/
Depression	0.708, *p* = 0.548	0.773, *p* = 0.510	/	/
Age	0.952, *p* = 0.384	0.358, *p* = 0.784	/	/

*Note:* Levene's test based on median and with adjusted *df*; 1 = Dangerous Self‐managers, 2 = Effective Self‐managers, 3 = Ambivalent empowered, 4 = Disempowered. *p < 0.05; ns = nonsignificant

** *p* < 0.01

*** *p* < 0.001.

## 7 Discussion

The study yielded compelling evidence that individuals who turn to OCMHs for assistance with mental health exhibit significant heterogeneity. As a first outcome, the cluster analysis unveiled the presence of four distinct clusters. Two of these (Dangerous self‐managers and Effective self‐managers) corresponded to the homonymous categories distinguished by Schulz and Nakamoto [34] in the Health Empowerment Model. With respect to the first cluster, the Dangerous self‐managers, it exhibited low levels of literacy, and overall low levels of patient empowerment, akin to the cluster of the Disempowered, with the exception of the Self‐determination dimension. The cluster, aligning with what was theorized by Schulz and Nakamoto [34], was later found in other studies in the field, albeit not within the context of mental health [30, 68, 70]. Paradoxically, the Dangerous self‐managers appeared to lack the adequate knowledge necessary for making informed health decisions while simultaneously assuming an authoritative role in their (mental) health management. According to the Health Empowerment Model [34], they might display a propensity to engage in non‐beneficial health behaviours such as medication adherence [32], participation in healthcare [109] or disease management [30, 110], potentially resulting in adverse health outcomes. This was confirmed in our study as they exhibited less favourable attitudes towards seeking professional help.

A cluster displaying an opposite pattern is one of the effective self‐managers, comprising roughly 20% of the participants, who had overall high levels of literacy and patient empowerment, displaying very positive attitudes towards professional help‐seeking, the highest together with the Disempowered. This strongly indicates that individuals within this cluster utilize OCMHs to further augment their health outcomes rather than merely supplementing traditional therapy.

The profile of the Disempowered aligns with findings from other studies, which noted that some members of online communities may exhibit lower levels of empowerment [76, 77, 78]. This cluster was the most prevalent (40%) and can be associated with what Schulz and Nakamoto [34] have defined as ‘Needlessly dependent patients’—a category composed of individuals with high levels of literacy but low empowerment, and as consequence, ‘highly dependent on health professionals despite their ability to make well‐informed decisions for themselves’ [143]. This cluster may indeed be more inclined to heavily rely on mental health professionals, and this is supported by their positive help‐seeking attitudes, not dissimilar to those exhibited by the Effective self‐managers. Taking into account that values of empowerment were measured using a 7‐point Likert scale, their scores for Meaning (3.08) and Impact (3.39) were slightly below the mean, suggesting that they still find significance in seeking and engaging with online information for their health. However, with respect to Competence (2.67) and Self‐determination (1.41), the values were quite scant, suggesting that these members place very low confidence in their own competence and self‐determination when it comes to healthcare decisions. Future studies are needed to determine whether this cluster is composed of disempowered, OCMHs sceptical, or underconfident patients.

The last category, those of the Ambivalent empowered (27.1%), shares similarities with the Dangerous self‐managers in terms of the lowest level of depression literacy, the second highest misinformation agreement and an overall high level of empowerment, albeit slightly lower levels in the Self‐determination dimension. It is important to mention that their agreement with correct information did not differ from the clusters of Effective empowered and Disempowered. In terms of their attitudes, they closely align with the negative attitudes observed in the Dangerous self‐managers cluster.

Additionally, the study sheds light on another critical outcome: there were differences in help‐seeking attitudes based on the identified clusters, even after controlling for depression and anxiety severity, for which no differences were observed across clusters. The Dangerous self‐managers cluster exhibited the most negative attitudes towards help‐seeking. They were followed by Ambivalent empowered which differed from both the Disempowered and Effective self‐managers clusters, the latter two exhibiting the most positive attitudes. Overall, it is evident that literacy played a significant role in shaping attitudes, as the two clusters with the highest overall literacy also displayed the most positive attitudes. This finding is consistent with previous literature in the field [84]. What is particularly interesting is that the two clusters with the most positive attitudes had exactly opposite patterns in terms of empowerment. In contrast, those with the most negative attitudes shared the common trait of having low depression literacy but felt capable of making autonomous decisions for their mental health (Self‐determination empowerment).

The fact that there were no differences in the severity of depression and anxiety among the identified clusters is particularly worrying, as individuals with more negative attitudes towards mental health treatment exhibited the same severity levels as the others. Furthermore, we found no differences in the share of participants not in treatment within the clusters (around 20%). However, the presence of more negative attitudes in certain clusters remains concerning, as it could deter not only future help‐seeking behaviour but also lower adherence to current psychological or pharmacological therapies. The phenomenon of non‐adherence to treatment programs and early withdrawal from mental healthcare is, in fact, not uncommon [111, 112, 113, 114, 115]. The identified reasons for dropout include, at this point non‐surprisingly, attitudinal barriers, self‐reliance and the belief that the problem will resolve on its own [116].

Regarding the second hypothesis, the study provides evidence that accounting for the multidimensionality of patient empowerment provides more nuanced insights into the individuals' typologies and how these interrelate with literacy. Specifically, the study identifies two distinct patterns: those with high Meaning empowerment, also had high levels of Competence and Impact. Self‐determination was instead a dimension kept as distinguished. The first three types of empowerment are in fact are rooted in motivational/volitional aspects. Self‐determination instead is more behaviourally oriented and concerning self‐reliance in mental health decision‐making.

To conclude, the study advances that knowledge about depression does not necessarily equate with general literacy about mental health, demonstrating that individuals can possess knowledge about depression while simultaneously holding misconceptions.

### 7.1 Practical Implications

The heterogeneity of members within OCMHs, as revealed through the cluster analysis, underscores the need for communities' administrators and platform designers to consider tailored approaches to ameliorate the content provided and to optimize users' experience (see Table 5).

**Table 5:** Patterns of clusters and potential interventions.

Clusters	Depression literacy	General mental health literacy*	Motivation‐based empowerment	Self‐determination empowerment	Help‐ seeking attitudes	Potential intervention
Dangerous self‐managers	↓	↓	↓	↑	**—**	↑ Health literacy ↑ Motivation‐based empowerment
Effective self‐managers	↑	↑	↑	↑	**+**	Sustain empowerment
Ambivalent empowered	↓	x̄	↑	↑	x̄	↑ Health literacy
Disempowered	↑	↑	↓	↓	**+**	↑ Overall empowerment

* Agreement with misinformation + agreement with correct information. ↓ = low levels, ↑ = high levels, x̄ = average levels, + = positive attitudes, − = negative attitudes.

Recognizing that health literacy is not a fixed asset but that rather it can be improved through education [36], we suggest the integration of mental health literacy education either prior to users accessing OCMHs or during their initial interactions with the platform. This also as literacy can confer strong resistance to health misinformation [117], an impending peril in social media [22]. The enhancement of literacy might also help to temper the perceptions of autonomy of the Dangerous self‐managers or the Ambivalent empowered. A recent meta‐analysis has highlighted the effectiveness of digital interventions in enhancing mental health literacy through diverse formats, such as structured programs with professionals or video‐based educational materials and illustrations both in websites or apps [118].

For the other clusters, such as the Disempowered, the recommendation is to strengthen their patient empowerment. This can be achieved through (i) interventions targeted directly at patients [119] or (ii) as a result of an interactive process [120, 121, 122] where clinicians, OCMH administrators and policymakers should create conditions [2, 123]. The first approach, building on the study results and guided by the Health Empowerment model [34], could involve interventions targeting specific dimensions of patients' perceptions of empowerment [124]. Digital interventions aimed at empowerment, such as workshop discussions upon hypothetical scenarios [125], have been proven effective [29, 125, 156].

These interventions, upon approval of the OCMH administrators, could be shared within the communities, with participants selected based on mental health literacy and empowerment level eligibility. Interventions could be designed as general mental health programs or disease‐specific content [126, 127].

Furthermore, both literacy and empowerment interventions could capitalize on the existing affordances of OCMHs. For instance, Facebook platforms already offer features such as Livestream events within Facebook groups [128], which could serve as effective channels for delivering interventions. A blended approach, combining digital interventions with clinical triage [129], could also be adopted.

However, it is crucial to emphasize that for groups such as Dangerous self‐managers and Ambivalent empowered, interventions should simultaneously improve empowerment and literacy as patient empowerment without adequate health literacy can lead to harmful consequences.

### 7.2 Limitations and Future Research

Our study has several limitations that should be taken into account. First, the cross‐sectional nature of our data does not allow any speculation regarding causality. A second limitation pertains to the measurement of patient empowerment as some of the dimensions consisted of only two items, which may have affected their internal consistency. A third aspect concerns the limited scope of the health literacy measurement: our assessment concerned declarative knowledge, overlooking aspects such as judgement skills and procedural help‐seeking knowledge.

Fourth, while we explored the interplay between patient empowerment and health literacy in predicting attitudes towards professional help‐seeking, these factors represent only part of the relevant variables influencing help‐seeking behaviour [24, 62, 130]. For example, systematic reviews [65, 67] have highlighted the importance of past experiences with mental health services in shaping help‐seeking behaviours. Future research should delve deeper into the interplay between mental health literacy, patient empowerment, help‐seeking attitudes and past experiences. For instance, individuals with low health literacy and negative past experiences with health professionals may become Dangerous self‐managers, relying on high levels of self‐determination empowerment as a compensatory strategy for perceived inadequacies in professional care and scepticism towards traditional therapies. However, their negative perception of healthcare may have also emerged as a consequence of low literacy [131, 132, 133, 134, 135] or patient empowerment [136, 137, 138], as other studies suggest.

Conversely, individuals with low literacy who have experienced positive experiences with healthcare providers may become overly reliant on professionals, resulting in a Disempowered (or Needlessly dependent) patient profile. These individuals may lack the confidence to make autonomous health decisions because their supportive experiences reinforce dependence rather than fostering empowerment. Alternatively, their low self‐confidence might have driven their initial help‐seeking behaviour (and related positive help‐seeking attitudes), believing they were incapable of managing health issues independently.

The intricate interactions among the aforementioned variables underscore the necessity for future longitudinal panel studies employing methodologies like cross‐lagged models [139] to disentangle the directionality of these causal relationships over time. Qualitative research—such as interviews exploring health attitudes and experiences [140]—could also provide valuable insights by exploring the lived experiences of individuals within identified clusters, offering a deeper understanding of their attitudes towards healthcare and how these are shaped by their personal and recovery histories. Additionally, past experiences are not limited to direct interactions with healthcare systems. As Brown et al. [141, p. 454] argue, ‘where there is little or no prior direct experience of mental health services, other bases such as social networks, the media, or previous experience with other health, welfare or state institutions may be drawn upon’.

## 8 Conclusions

The present study had as a context online communities for mental health. Clusters created from the interaction of mental health literacy and patient empowerment resulted in significant differences in professional help‐seeking attitudes. The study provides further evidence of the multidimensionality of patient empowerment. In light of these findings, this research paves the way for the development of targeted interventions that acknowledge the unique characteristics faced by individuals within each cluster.

## Author Contributions

**Nicole Bizzotto:** conceptualization, investigation, funding acquisition, writing–original draft, methodology, visualization, writing–review and editing. **Gert‐Jan Bruijn:** supervision. **Peter Johannes Schulz:** supervision.

## Conflicts of Interest

The authors declare no conflicts of interest.

## Supporting information

Supporting information.

## Data Availability

The data that support the findings of this study are available on request from the corresponding author.
