# Supplementary material for: Clusters of Patient Empowerment and Mental Health Literacy Differentiate Professional Help‐Seeking Attitudes in Online Mental Health Communities Users
Source: Health Expect. 2025 Jan 15;28(1):e70153. doi: 10.1111/hex.70153 (PMC11735741; doi:10.1111/hex.70153)
Supplement: Supplementary file 1 — Supporting information. [file HEX-28-e70153-s001.docx]

**Supplement**

**Clusters of Patient Empowerment and Mental Health Literacy Differentiate Professional Help‐Seeking Attitudes in Online Mental Health Communities Users**

**Patient Empowerment in**

**Online Mental Health Communities Scale**

**Response Options:** A 7-point Likert scale ranging from 1 (Strongly Disagree) to 7 (Strongly Agree).

***Empowerment as Meaningfulness (3 items)***

1. I find that engaging in mental health-themed Facebook groups helps me better cope with my illness.
2. Participation in mental health-themed Facebook groups improves both my health and overall well-being.
3. Engaging in mental health-themed Facebook groups is aiding me in managing my condition more effectively.

***Empowerment as Competence (3 items)***

1. Mental health-themed Facebook groups have given me a deeper understanding of my condition.
2. Through my participation in these groups, I feel better prepared for appointments with mental health professionals.
3. Thanks to mental health-themed Facebook groups, I am better able to identify the best treatment options for myself.

***Empowerment as Self-determination (2 items)***

1. Participation in mental health-themed Facebook groups has made me realize that I can decide when and whether to stop medication or psychotherapy.
2. Participation in mental health-themed Facebook groups has made me realize that I can make my own decisions regarding my mental health.

***Empowerment as Impact (2 items)***

1. Participation in mental health-themed Facebook groups made me realize that I can decide between different treatment options for my illness.
2. Participation in mental health-themed Facebook groups made me realize that my mental health also depends on my commitment.
